# Liver transplantation for Type I Abernethy malformation with liver tumors in children: a report of two cases with contrasting portal vein reconstruction strategies

**DOI:** 10.1186/s12887-026-07044-6

**Published:** 2026-05-23

**Authors:** Huanli Han, Mingman Zhang, Xiaoke Dai

**Affiliations:** 1 Department of Pediatric Hepatobiliary Surgery, Children’s Hospital of Chongqing Medical University, Chongqing, 400014 P.R. China; 2 National Clinical Research Center for Children and Adolescents’ Health and Diseases, Ministry of Education Key Laboratory of Child Development and Disorders, International Science and Technology Cooperation Base of Child Development and Critical Disorders, Chongqing Key Laboratory of Structural Birth Defect and Reconstruction, Chongqing, 400014 P.R. China

**Keywords:** Abernethy malformation, Liver transplantation, Congenital portosystemic shunt, Children, Portal vein reconstruction

## Abstract

**Background:**

Type I Abernethy malformation is a rare congenital portosystemic shunt with an absent intrahepatic portal vein (PV). It often presents with liver tumors or metabolic disorders. Liver transplantation (LT) is the only definitive treatment, but it poses unique technical challenges due to the absent native PV. Detailed surgical guidance for variable anatomies remains scarce.

**Case presentation:**

We report two children with Type I Abernethy malformation who underwent LT in 2023. Both presented with benign liver tumors, hyperammonemia and abnormal liver function. Case 1 (2.5 y/m) received a deceased-donor left-lobe LT. Owing to insufficient PV length, a deceased-donor iliac vein graft was used for PV reconstruction. Case 2 (5.7 y/f) underwent living-donor left-lateral-sector LT with direct anastomosis of the donor left PV to the recipient’s shunt vessel. Graft-to-recipient weight ratios (GRWR) were 2.29% and 1.45%, respectively.

**Results:**

Both transplant procedures were successful. Postoperative liver function and ammonia normalized. Case 2 developed hepatic venous outflow obstruction (HVOO) postoperatively, which was successfully managed with balloon angioplasty on POD 24. At 18-month follow-up, both patients were alive with good graft function, without tumor recurrence or metabolic abnormalities. To our knowledge, this is among the few reports with 18-month follow-up for pediatric patients with this disease after LT using these techniques.

**Conclusion:**

This report offers a practical and anatomy-based surgical framework for PV reconstruction in pediatric Type I Abernethy malformation: direct anastomosis when an adequate shunt vessel is present, or interposition grafting (e.g., with iliac vein) when the native portal stump is absent. These contrasting strategies achieved good early outcomes at 18 months, suggesting that LT can be a curative option for this challenging anomaly. These observations are primarily hypothesis-generating. Validation will require larger, multicenter cohorts.

## Introduction

Type I Abernethy malformation is a rare congenital portosystemic shunt in which the intrahepatic portal vein (PV) is absent and portal flow is completely diverted to the systemic circulation [1]. This anatomy inevitably leads to two interrelated pathologies. First, portal deprivation causes hepatic nodules or tumors, including adenoma, focal nodular hyperplasia, and rarely malignancy [2]. Second, systemic metabolic derangements occur, most notably hyperammonemia with potential neurocognitive impairment [3]. Non-transplant interventions (tumor resection or shunt occlusion) fail to correct the underlying pathophysiology and may precipitate hepatic failure [4]. Liver transplantation (LT) restores physiological portal inflow and removes the native liver at risk, thus offering a definitive cure.

However, LT in this setting poses a specific technical challenge: how to reconstruct portal inflow without a native PV stump. Published reports are limited to isolated cases and lack a structured surgical approach. We describe two pediatric patients with Type I Abernethy malformation and concomitant liver tumors. They received LT with contrasting and anatomy-driven PV reconstruction strategies. Detailed reports of iliac vein interposition grafting for PV reconstruction in a young child with this anomaly are few. This report provides a detailed description of the technique. We aim to provide a preliminary and anatomy-based reference for surgical teams planning LT in this rare and anatomically challenging disorder.

## Case presentations

### Case 1: interposition graft reconstruction

**Fig. 1:**
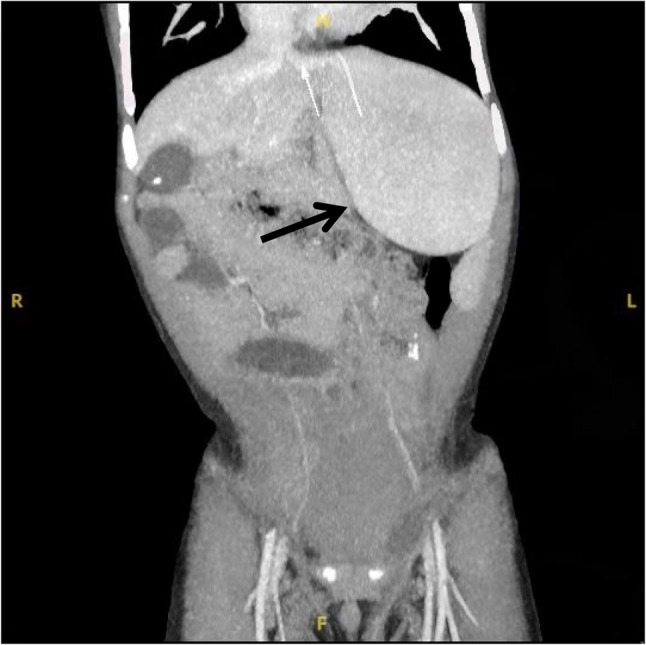
A large (approximately 6.2 × 8.3 × 8.8 cm) mass is visible in the left hepatic lobe (black arrow). The nature of the mass was unclear; liver biopsy confirmed hepatocellular adenoma

**Fig. 2:**
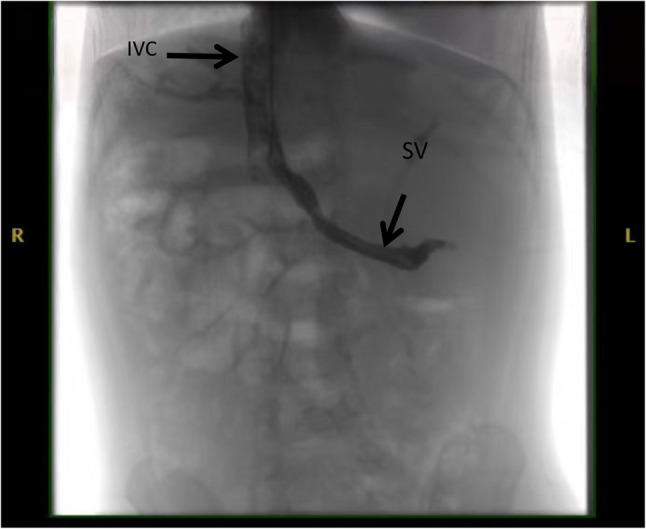
DSA shows that blood from the portal system does not enter the liver, and the shunt vessel drains directly into the inferior vena cava (IVC)

**Fig. 3:**
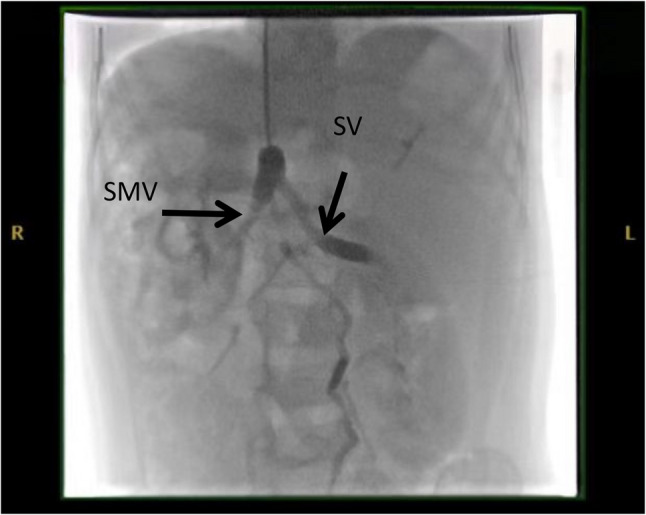
Intraoperative DSA following temporary balloon occlusion of the portosystemic shunt at its confluence with the inferior vena cava (IVC). The splenic vein (SV) and superior mesenteric vein (SMV) converge to form the shunt vessel. The intrahepatic portal vein is not visualized, confirming its congenital absence

A 2.5-year-old male presented with a liver mass. Laboratory investigations revealed elevated liver enzymes (ALT 69 U/L, AST 106 U/L), hyperbilirubinemia (direct bilirubin 16.2 µmol/L), and hyperammonemia (64.7 µmol/L). Serum alpha-fetoprotein (AFP) was elevated at 55.8 ng/mL (normal < 6 ng/mL). Computed tomography (CT) showed a large left hepatic lobe mass, about 6.2 × 8.3 × 8.8 cm (Fig. 1). Digital subtraction angiography (DSA) confirmed Type I Abernethy malformation with absent main PV. The splenic vein (SV) and superior mesenteric vein (SMV) converged to form a shunt vessel that drained directly into the inferior vena cava (IVC) (Figs. 2 and 3). The biopsy confirmed hepatocellular adenoma.

**Fig. 4:**
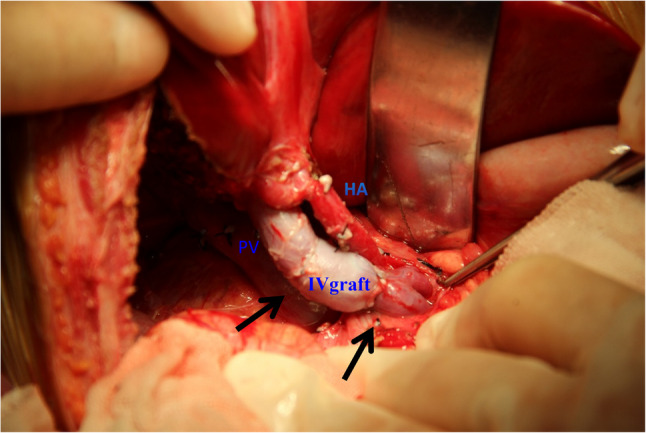
Complex portal vein reconstruction in Case 1. The intraoperative photograph shows portal vein reconstruction using an interposition deceased-donor iliac vein graft (the vascular segment between the two black arrows). The black arrow 1 indicates the proximal anastomosis (donor left portal vein to iliac vein graft); black arrow 2 indicates the distal anastomosis (iliac vein graft to the recipient’s large-diameter shunt vessel). HA: hepatic artery; PV: portal vein. IV graft: iliac vein graft

He underwent piggyback orthotopic LT using a deceased-donor left lobe graft (GRWR 2.29%). Given the complete absence and insufficient length of the native PV for direct anastomosis, PV reconstruction was achieved using an interposition graft. A segment of deceased donor right iliac vein was anastomosed to the donor left PV branch proximally and to the recipient’s portosystemic shunt vessel distally (Fig. 4). Arterial reconstruction involved anastomosing the donor left hepatic artery to the recipient’s proper hepatic artery.

### Case 2: direct anastomosis

**Fig. 5:**
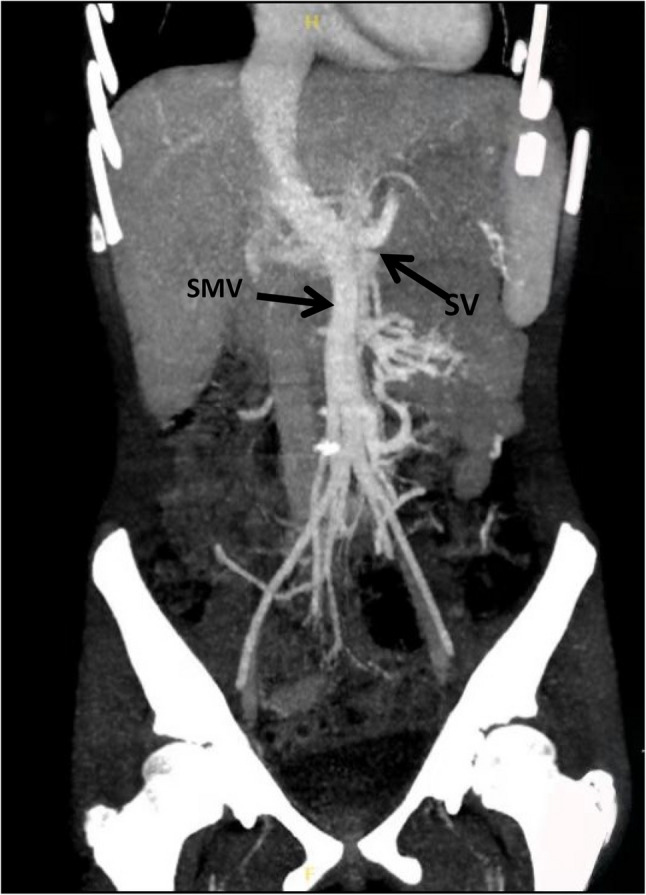
CT showed that the superior mesenteric vein (SMV) and splenic vein confluence (SV) joined the trunk and drained into the inferior vena cava (IVC); the portal vein was not shown

A 5.7-year-old female presented with multiple liver masses and a history of recurrent headaches, inattention, and declining academic performance. Laboratory tests showed elevated transaminases (ALT 97 U/L, AST 80 U/L) and hyperammonemia (105 µmol/L). AFP was normal. CT demonstrated multiple liver masses, and the largest located in the left lateral lobe ( 4.2 × 4.3 cm). The SMV and SV converged into the IVC (Fig. 5). DSA confirmed Type I Abernethy malformation, revealing a 10 mm shunt vessel from the confluence to the IVC. The biopsy showed focal nodular hyperplasia (FNH).

**Fig. 6:**
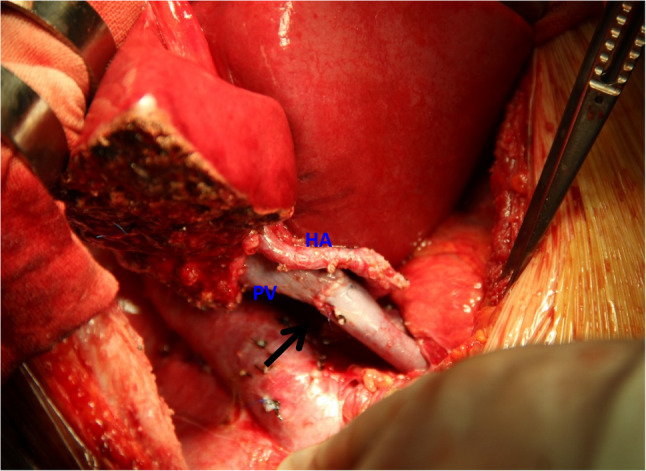
Direct portal vein anastomosis in Case 2. The intraoperative photograph shows the end-to-end anastomosis between the donor left portal vein (PV) and the recipient’s large-diameter shunt vessel. The black arrow indicates the anastomotic site. HA: hepatic artery; PV: portal vein

She underwent piggyback orthotopic LT with a living-donor left lateral lobe graft (GRWR 1.45%). The well-developed shunt vessel enabled direct end-to-end anastomosis between the donor left PV and the recipient’s shunt vessel (Fig. 6). Arterial reconstruction was performed by anastomosing the donor left hepatic artery to the recipient’s right hepatic artery.

### Outcomes

Both transplant procedures were completed successfully. The clinical characteristics, surgical details, and postoperative outcomes are summarized in Table 1. Serial postoperative liver function tests are shown in Table 2.

**Table 1:** Clinical characteristics, surgical details, and outcomes of two pediatric patients with Type I Abernethy malformation

Parameter	Case 1	Case 2
Demographics		
Age/Sex	2.5y / Male	5.7y / Female
Presenting Symptoms	Large liver mass	Multiple liver mass, headaches, poor attention
Preoperative Data		
Liver Tumor Type	Hepatocellular adenoma	Focal nodular hyperplasia (multiple)
Preoperative AFP (ng/mL)	55.8	Within normal range
Preoperative Ammonia (µmol/L)	64.7	105
Transplantation Details		
Graft Type	Deceased donor left lobe	Living donor left lateral lobe
GRWR (%)	2.29	1.45
Transplantation Technique	Piggyback orthotopic	Piggyback orthotopic
Portal Vein Reconstruction	Right iliac vein graft interposition	Direct anastomosis (donor LPV to shunt)
Operative Time (h)	9	10
Anhepatic Phase (min)	81	75
Blood Loss (mL)	400	400
Postoperative Course		
Complication	None	*HVOO*
Management	-	Balloon angioplasty on POD 24
Hospital Stay (days)	30	29
Follow-up (18 months)		
Graft Function	Normal	Normal
Tumor Recurrence	No	No
Growth Status	50th–75th percentile	25th–50th percentile

*Abbreviations*: *AFP* Alpha-fetoprotein, *GRWR* Graft-to-recipient weight ratio, *LPV* Left portal vein, *HVOO* Hepatic venous outflow obstruction, *POD* postoperative day

Normal reference ranges: AFP: <6 ng/mL (for age); Ammonia: 9–30 µmol/L

**Table 2:** Serial postoperative liver function tests

Postoperative Day	Case 1 – Direct Bilirubin (µmol/L)	Case 1 – ALT (U/L)	Case 2 – Direct Bilirubin (µmol/L)	Case 2 – ALT (U/L)
POD 1	44.4	1201	48	473
POD 3	31.8	565	32.5	245
POD 7	8.9	109	4.8	165
POD 14	4.8	34	5.1	146
POD 21	2.7	17	2.7	96
POD 28	3.0	19	3.6	52
Normal Range	0–10	11–45	0–10	11–45

In Case 1, direct bilirubin normalized by POD 6, and ALT by POD 13. In Case 2, direct bilirubin normalized by POD 6. ALT declined from 473 U/L (POD 1) to 165 U/L (POD 7) but plateaued between POD 7–14. The patient developed increased pleural effusion and ascites around POD 10. Doppler ultrasound suggested hepatic venous outflow obstruction (HVOO), which was confirmed by percutaneous hepatic venography on POD 24 (Clavien-Dindo grade IIIb). Balloon angioplasty immediately resolved the stenosis. ALT fell to 52 U/L by POD 28, and effusion and ascites resolved. The patient was discharged on POD 29.

Both patients achieved complete metabolic correction, with blood ammonia levels returning to and remaining within the normal range postoperatively. At 18 months post-transplant, both were alive with excellent graft function, normal mental status, unrestricted activity, and no tumor recurrence or metabolic abnormalities. No HVOO recurrence occurred in Case 2.

## Discussion

This report offers a preliminary and anatomy-based decision framework for PV reconstruction in pediatric LT for Type I Abernethy malformation. It contrasts interposition grafting with direct anastomosis. Few detailed reports exist on iliac vein interposition grafting for PV reconstruction in a young child with this anomaly. Our report provides an 18-month follow-up, making it one of the few reports with this follow-up duration for this condition.

### Transplant indications

Partial hepatectomy does not correct portosystemic shunting and leaves the residual liver at risk for tumor recurrence or de novo formation [5]. Shunt occlusion is contraindicated because it can cause acute mesenteric venous hypertension and intestinal ischemia [4]. Thus, LT is a therapy that corrects both the shunt and its hepatic sequelae.

Our two cases illustrate distinct indications. Case 1 had a large, high-risk adenoma with elevated AFP. Case 2 had multiple FNH and symptomatic hyperammonemia with neurocognitive decline, and halting progressive deterioration was the primary goal.

### Individualized PV reconstruction: the surgical challenge and technical cornerstone

The principal technical challenge in LT is reconstructing portal inflow without a native PV. Our experience demonstrates that success is not contingent on a single technique, but on individualized strategies formulated through preoperative radiological evaluation. Severe foreshortening of the native portal structures in Case 1 necessitated innovative reconstruction. The gap between the donor left PV and the recipient’s shunt vessel was bridged using a deceased donor right iliac vein graft as an interposition conduit. By contrast, Case 2 had a long, well-developed shunt vessel, which enabled a direct end-to-end anastomosis between the donor left PV and the recipient’s shunt. These two contrasting strategies illustrate a practical and anatomy-based decision framework: interposition grafting when the shunt vessel is inadequate for direct anastomosis, and direct anastomosis when anatomy permits. This framework is especially valuable when tension-free reconstruction is not possible. It helps guide the surgeon to the appropriate conduit choice.

GRWR differed between our cases: 2.29% in Case 1 versus 1.45% in Case 2. Neither developed small-for-size syndrome (SFSS). While this observation generates only hypotheses, it suggests that a lower GRWR may be acceptable when a tension-free and anatomically favorable portal anastomosis is achieved. However, GRWR thresholds derived from cirrhotic patients may not directly apply to this unique physiology. Graft selection should primarily be guided by anatomical feasibility rather than GRWR alone [6].

### Complication management

Case 2 was complicated by HVOO, which occurs in 3%–9% of piggyback LTs [7]. Clinical suspicion should arise from refractory ascites/pleural effusion or cholestatic graft dysfunction, prompting timely Doppler ultrasound screening. Percutaneous hepatic venography is the gold standard for definitive diagnosis, and balloon angioplasty is the first-line treatment [8]. Evidence-based technical modifications, including triangular or interrupted anastomotic techniques, might further reduce HVOO incidence in left lateral segment grafts [9, 10].

### Limitations and clinical implications

Several limitations warrant explicit acknowledgment. First, with only two cases, we cannot perform statistical inference, and the generalizability of our findings is severely limited. This limitation is unavoidable given the extreme rarity of the condition. Second, this is a single-center retrospective report, which carries inherent risks of selection bias and information bias. Third, this 18-month follow-up reflects only early results. Long-term graft survival, metabolic stability, and quality of life still need to be assessed. Given these limitations, our findings generate hypotheses rather than providing definitive answers.

However, the anatomy-based decision framework we describe provides a practical reference for surgeons facing the rare but challenging scenario. Our experience shows that early transplantation is critical for preventing potentially irreversible neurocognitive decline from chronic hyperammonemia and for avoiding malignant transformation or hemorrhage in hepatic adenomas, especially large ones. Children with confirmed Type I Abernethy malformation and clinically relevant hepatic lesions or metabolic complications warrant prompt referral to a high-volume pediatric liver transplant center. Future studies with larger, multicenter cohorts are needed to standardize indications, refine surgical techniques, and establish robust long-term outcome benchmarks.

## Conclusion

In children with Type I Abernethy malformation complicated by liver tumors or metabolic disorders, LT offers a definitive cure. Success depends on anatomy-driven, individualized PV reconstruction: direct anastomosis when an adequate shunt vessel is present, or interposition grafting (e.g., with iliac vein) when the native portal stump is absent. Our 18-month outcomes are encouraging, and this preliminary anatomy-based strategy provides a practical reference for surgical teams. Given the rarity of this disease, international collaboration will therefore be important to validate these observations.

## Data Availability

No datasets were generated or analysed during the current study.
